# Splicing factor-mediated regulation patterns reveals biological characteristics and aid in predicting prognosis in acute myeloid leukemia

**DOI:** 10.1186/s12967-022-03868-9

**Published:** 2023-01-07

**Authors:** Fang-Min Zhong, Fang-Yi Yao, Jing Liu, Mei-Yong Li, Jun-Yao Jiang, Ying Cheng, Shuai Xu, Shu-Qi Li, Nan Zhang, Bo Huang, Xiao-Zhong Wang

**Affiliations:** 1 Jiangxi Province Key Laboratory of Laboratory Medicine, Jiangxi Provincial Clinical Research Center for Laboratory Medicine, Department of Clinical Laboratory, The Second Affiliated Hospital of Nanchang University, Nanchang, Jiangxi China; 2 School of Public Health, Nanchang University, No. 461 BaYi Boulevard, Nanchang, 330006 Jiangxi China

**Keywords:** Alternative splicing, Splicing factor, Tumor microenvironment, Prognosis, SRSF10

## Abstract

**Background:**

Alternative splicing (AS) of RNA is a fundamental biological process that shapes protein diversity. Many non-characteristic AS events are involved in the onset and development of acute myeloid leukemia (AML). Abnormal alterations in splicing factors (SFs), which regulate the onset of AS events, affect the process of splicing regulation. Hence, it is important to explore the relationship between SFs and the clinical features and biological processes of patients with AML.

**Methods:**

This study focused on SFs of the classical heterogeneous nuclear ribonucleoprotein (hnRNP) family and arginine and serine/arginine-rich (SR) splicing factor family. We explored the relationship between the regulation patterns associated with the expression of SFs and clinicopathological factors and biological behaviors of AML based on a multi-omics approach. The biological functions of SRSF10 in AML were further analyzed using clinical samples and in vitro experiments.

**Results:**

Most SFs were upregulated in AML samples and were associated with poor prognosis. The four splicing regulation patterns were characterized by differences in immune function, tumor mutation, signaling pathway activity, prognosis, and predicted response to chemotherapy and immunotherapy. A risk score model was constructed and validated as an independent prognostic factor for AML. Overall survival was significantly shorter in the high-risk score group. In addition, we confirmed that SRSF10 expression was significantly up-regulated in clinical samples of AML, and knockdown of SRSF10 inhibited the proliferation of AML cells and promoted apoptosis and G1 phase arrest during the cell cycle.

**Conclusion:**

The analysis of splicing regulation patterns can help us better understand the differences in the tumor microenvironment of patients with AML and guide clinical decision-making and prognosis prediction. SRSF10 can be a potential therapeutic target and biomarker for AML.

**Supplementary Information:**

The online version contains supplementary material available at 10.1186/s12967-022-03868-9.

## Introduction

Acute myeloid leukemia (AML) is a hematological malignancy derived from hematopoietic stem cells, and its pathogenesis is still unclear [1]. Patients with AML have a very poor prognosis, with a 5-year survival rate lower than 30% [2]. The “7 + 3” chemotherapy with cytarabine and anthracyclines is the conventional clinical treatment for AML [3]. Targeted therapies for gene mutations, such as FLT3 and IDH, and apoptosis-inducing therapies for BCL-2 have also been developed successively [4, 5]. However, due to the intolerability of chemotherapy and the emergence of treatment resistance, the treatment of AML is difficult. Therefore, it is very important to thoroughly study the molecular biological characteristics of AML and explore new therapeutic targets.

Aberrant splicing of genes promotes AML progression as well as treatment resistance, including the formation of various specific splice variants and the onset of non-characteristic alternative splicing (AS) events [6, 7]. These aberrant tumor alterations can adequately create self-serving survival conditions, evade attack by the immune system, or induce aberrant energy metabolism. For example, splice variants of *NOTCH2* and *FLT3* genes encode functional proteins that exert pro-oncogenic effects and generate resistance against targeted inhibitors by affecting key downstream signaling targets (AKT, STAT, and ERK) [8]. Among anti-apoptotic BCL-2 proteins, MCL-1 L can promote AML cell survival by isolating the pro-apoptotic proteins BIM and BID [9]. In AML, the production of functional protein products with oncogenic properties is largely dependent on the regulation of transcript expression levels, and aberrant alterations in pro-tumor cell survival pathways, oncogenic transcription factors, intrinsic and extrinsic apoptotic signaling, and death effector molecules can be mediated by splicing regulation to establish chemoresistant phenotypes [10, 11].

AS is a complex biological process that can involve coordinated interactions between more than 200 molecules to support or inhibit splicing regulation at specific target sites of precursor messenger RNA (pre-mRNA) [12]. In this process, splicing factors (SFs) play an important role, which are proteins that form part of a dynamic complex called the spliceosome with at least five small nuclear RNAs (snRNAs) [13]. Like “scissors”, it precisely repairs the pre-mRNA, cuts off the redundant part to form a variety of mRNA sequences, and then translates to form protein isoforms with different biological functions, participating in the life activities of the whole body [14]. There are two well-known protein families in RNA-binding SFs: serine/arginine-rich (SR) proteins, which normally promote exon inclusion, and heterogeneous nuclear ribonucleoproteins (hnRNPs), which normally promote exon skipping [15]. In hematological tumors, more than half of patients with myelodysplastic syndromes (MDS) show mutations in functional components of the spliceosome, commonly in serine-rich SFs, such as SF3B1, SRSF2, and U2AF1 [16]. However, mutations in SFs are uncommon in AML compared to those in MDS [17], and therefore, aberrant splicing regulation of SFs may play a more significant role in disease progression in AML. Events in AS may be an essential feature of AML biology, and genome-wide analysis of aberrant splicing patterns in patients with AML has revealed differential splicing of approximately one-third of genes in AML cells compared to those in CD34+ cells in a normal control population [18]. In two study cohorts comprising over 200 patients with AML, 135–786 genes that underwent recurrent splicing were identified per patient sample [18], of which approximately 76–80% of splicing changes could be mapped to translated transcriptional regions and potentially alter certain functions of proteins. Furthermore, changes occurring in non-translated regions could affect transcriptional stability or translation efficiency [18]. Approximately half of the identified splice variants have never been reported before, and these may be pathological factors specific to AML. However, in multiple patient samples, the presence and abundance of some splice variants could only be detected at diagnosis and then disappears during remission, only to be strongly re-expressed again during relapse.

This study aimed to conduct an in-depth analysis of the relationship between SFs of the hnRNP and SR families and the biological features of AML and to enhance the understanding of the mechanism by which these SFs regulate aberrant AS events in AML. These analyses may help us to better understand the role of AS pathogenesis on the development of AML. We applied bioinformatics analysis methods to transcriptome sequencing data combined with clinical information to explore the relationship among SFs, the prognosis of patients with AML, and tumor microenvironment. The results of these differential features and prognostic analysis will also provide reference values for the study of molecular mechanisms of AML, clinical prognosis prediction, and the design of personalized treatment regimens. Finally, we focused on the splicing factor *SRSF10*, a gene that plays an active tumor-promoting role in many cancers but whose expression has not been reported in AML. We observed that the expression levels of *SRSF10* were significantly upregulated in AML samples compared to those in normal blood tissues. Furthermore, we elucidated the mechanism of SRSF10 in AML via further experiments.

## Method

### Data acquisition and processing

We downloaded transcriptome sequencing data and clinical information from the XENA database (https://xenabrowser.net/datapages/) for 173 patient samples in the Cancer Genome Atlas-Acute Myeloid Leukemia (TCGA-LAML) and 337 healthy whole blood control samples in the Genome Tissue Expression (GTEx). The alternative splicing data in TCGA-LAML were downloaded in the TCGA SpliceSeq database, including the splicing percentage values (PSI) of alternative splicing events in each sample. PSI values ranging from 0 to 100% represented the occurrence of each AS event. Seven types of AS events were recorded, which are listed as follows: exon skipping (ES), mutually exclusive exon (ME), retained intron (RI), alternative promoter (AP), alternative terminator (AT), alternative donor site (AD), and alternative acceptor site (AA). Finally, we downloaded somatic mutation and copy number variation data from the TCGA database for patients with AML. RNA-seq data were normalized to transcripts per million (TPM) values and log2 transformed. Somatic mutation data and copy number variation data were downloaded from the TCGA database (https://portal.gdc.cancer.gov/). The microarray data and prognostic information of the validation cohorts for the risk score model (GSE14468, GSE37642-GPL96, GSE37642-GPL570, GSE71014) were downloaded from the Gene Expression Omnibus (GEO) database (https://www.ncbi.nlm.nih.gov/geo/). SFs from 20 HNRNP families and 12 SR families were analyzed in this study, which are listed as follows: SRSF1, SRSF2, SRSF3, SRSF4, SRSF5, SRSF6, SRSF7, SRSF8, SRSF9, SRSF10, SRSF11, SRSF12, HNRNPA0, HNRNPA1, HNRNPA2B1, HNRNPA3, HNRNPAB, HNRNPC, HNRNPCL1, HNRNPD, HNRNPDL, HNRNPF, HNRNPH1, HNRNPH2, HNRNPH3, HNRNPK, HNRNPL, HNRNPM, HNRNPR, HNRNPU, HNRNPUL1, and HNRNPUL2. All data were analyzed using the R language software R 4.02 and corresponding software packages.

### Unsupervised cluster analysis of 32 splicing factors

In order to better analyze the overall splicing regulation relationship of the 32 splicing factors, we used a consensus clustering algorithm to perform unsupervised clustering according to the expression of splicing factors for determining the splicing regulation patterns induced by the expression of splicing factors. The “ConsensuClusterPlus” package was used to perform the aforementioned steps, and this analysis was repeated 1000 times to ensure the stability of the clustering results. The t-Stochastic Neighbor Embedding (t-SNE) method was used to verify the reliability of the splicing regulation patterns identified based on SFs.

### Gene set variation analysis (GSVA) and functional annotation

In order to explore the differences in biological processes among different splicing regulatory patterns, we used the “GSVA” package to conduct GSVA enrichment analysis. In non-parametric and unsupervised analysis methods, GSVA is typically performed to calculate enrichment scores to estimate the activity levels of pathways and biological processes corresponding to gene sets in samples. We downloaded the “h.all.v7.4.symbols” gene set from MSigDB database (https://www.gsea-msigdb.org/gsea/msigdb/), and the adjusted P-value < 0.05 was used to identify the differences of enrichment scores among different groups. To explore the signaling pathways associated with differentially expressed genes (DEGs), we performed the Kyoto Encyclopedia of Genes and Genomes (KEGG) analysis. The biological functions of these genes were annotated via Gene Ontology (GO). These functions were analyzed in the“clusterProfiler” package. Gene sets for different types of immune-function related features, such as antigen-presenting cell co-stimulation/co-inhibition and T cell co-stimulation/co-inhibition, were collected from previous studies and then analyzed for enrichment scores using the GSVA algorithm [19].

### Estimation of immune cell infiltration in the tumor microenvironment

CIBERSORT uses a support vector regression algorithm to infer the proportion of immune cells in a tumor sample by deconvolution based on the expression of immune cell marker genes. These include naive B cells, memory B cells, plasma cells, CD8+ T cells, naive CD4+ T cells, resting CD4+ memory T cells, activated CD4+ memory T cells, follicular helper T cells, regulatory T cells, γδT cells, resting NK cells, activated NK cells, monocytes, M0 macrophages, M1 macrophages, M Macrophages, resting dendritic cells, activated dendritic cells, resting mast cells, activated mast cells, eosinophils, neutrophils and other 22 types of immune cells.

### Prediction of drug sensitivity and assessment of response to immunotherapy

The software package “pRRophetic” was used to predict the half-maximal inhibitory concentration (IC50) of drugs commonly used to treat AML in each sample. IC50 indicates the effectiveness of a substance in inhibiting a specific biological or biochemical function, and a smaller value indicates a better effect. The SubMap (s://cloud.genepattern.org/gp) algorithm was used to predict the response of different splicing regulatory patterns to anti-PD-1 and anti-CTLA4 immune checkpoint inhibitors.

### Construction of risk score model for prognostic prediction

Univariate Cox regression analysis was used to identify DEGs related to the prognosis of patients with different splicing regulation patterns, and LASSO Cox regression analysis was used to reduce the dimension to remove redundant prognosis-related molecules to prevent overfitting of the model. Furthermore, tenfold cross-validation was performed to determine the penalty parameter (λ) of the model. The following formula was used to calculate the risk score for each sample:

$$Risk\;score={\sum_1^i}(Coefi * ExpGenei),$$

where “Coef” represents the non-zero regression coefficient of the model gene, and “ExpGene” specifies the expression value of the model gene. All samples were divided into low- and high-risk groups according to the cut-off value of risk score. Kaplan–Meier analysis with log-rank test was used to compare differences in overall survival (OS) between low- and high-risk groups. We further plotted the time-dependent receiver operating characteristic (ROC) curve to evaluate the prognostic accuracy of the risk score model. Univariate and multivariate Cox analyses were used to determine the independent predictive power of the model.

### Development of a nomogram for predicting OS

In order to more accurately predict the OS of patients, we combined clinical features significantly associated with the prognosis of AML patients with the risk scoring model and developed a nomogram with three signatures: age, cytogenetic risk, and prognostic risk score model. Simultaneously, the ROC curve and calibration curve were drawn to evaluate the predictive performance of the nomogram.

### Collection of clinical samples and cell culture

Two batches of AML samples were collected with the approval of the Medical Ethics Committee of the Second Affiliated Hospital of Nanchang University, and the participants were informed at the same time. The first batch comprised bone marrow samples from four patients with AML and peripheral blood samples from eight healthy individuals. The second batch comprised peripheral blood samples from 22 patients with AML and 23 healthy individuals, and mononuclear cells were isolated. The mononuclear cells we obtained were left over from the necessary medical tests of the participants and would not affect their benefits. AML cell line THP1 was cultured in the RPMI1640 medium containing 10% fetal bovine serum and 1% penicillin–streptomycin in an incubator at 37 °C in a humidified atmosphere with 5% CO_2_. A lentivirus containing SRSF10 siRNA was purchased from Hanhsinhsinh (Shanghai, China) to infect THP1 cells and select cells for puromycin resistance. Real-time polymerase chain reaction (RT-PCR) was performed using a Japanese TAKARA kit using the ABI7500 instrument. Western blot experiments were performed with rabbit anti-β-tubulin (1:10,000, #2146) and anti-SRSF10 (1:1000, 42267S) antibodies purchased from Cell Signaling Technology (Danvers, MA, USA).

### Detection of cell proliferation, apoptosis, and cell cycle

Cell proliferation was detected by Cell Counting KIT-8 (CCK-8) and EdU staining. For CCK8, 2 × 10^4^ cells of different treatment groups were seeded in a 96-well plate, and each group was repeated three times. A total of 10 µL of CCK8 was added at 0, 24, 48, and 72 h, respectively. After incubation at 37 °C for 2.5 h, the OD was detected at 450 nm using a microplate reader. For EdU staining, 1 × 10^6^ cells were incubated with EdU solution diluted at a ratio of 1:1000 at 37 °C for 2.5 h, fixed with paraformaldehyde at room temperature, and decolorized with 2 mg/ml of glycine. After subsequent washing during Apollo staining and Hoechst33342 chamber staining, fluorescence microscopy was performed. For apoptosis assays, cells were stained using the annexin V-PE/7-AAD Apoptosis Assay Kit and subsequently analyzed via a flow cytometer. For the cell cycle, the cells were fixed with 75% ethanol precooled at 4 °C for 3–4 h and centrifuged at 1000 R for 5 min in a low-speed centrifuge. The supernatant was discarded and washed once with PBS. Subsequently, FITC dye was added, followed by incubation in a dark environment for about 15 min, and the cells were tested using a flow cytometer.

### Statistical analysis

We used the Wilcoxon rank sum test or the Kruskal–Wallis test to determine differences between groups. A two-sided P value of < 0.05 was considered statistically significant.

## Results

### The landscape of genetic variation in SFs of hnRNP and SR families

**Fig. 1:**
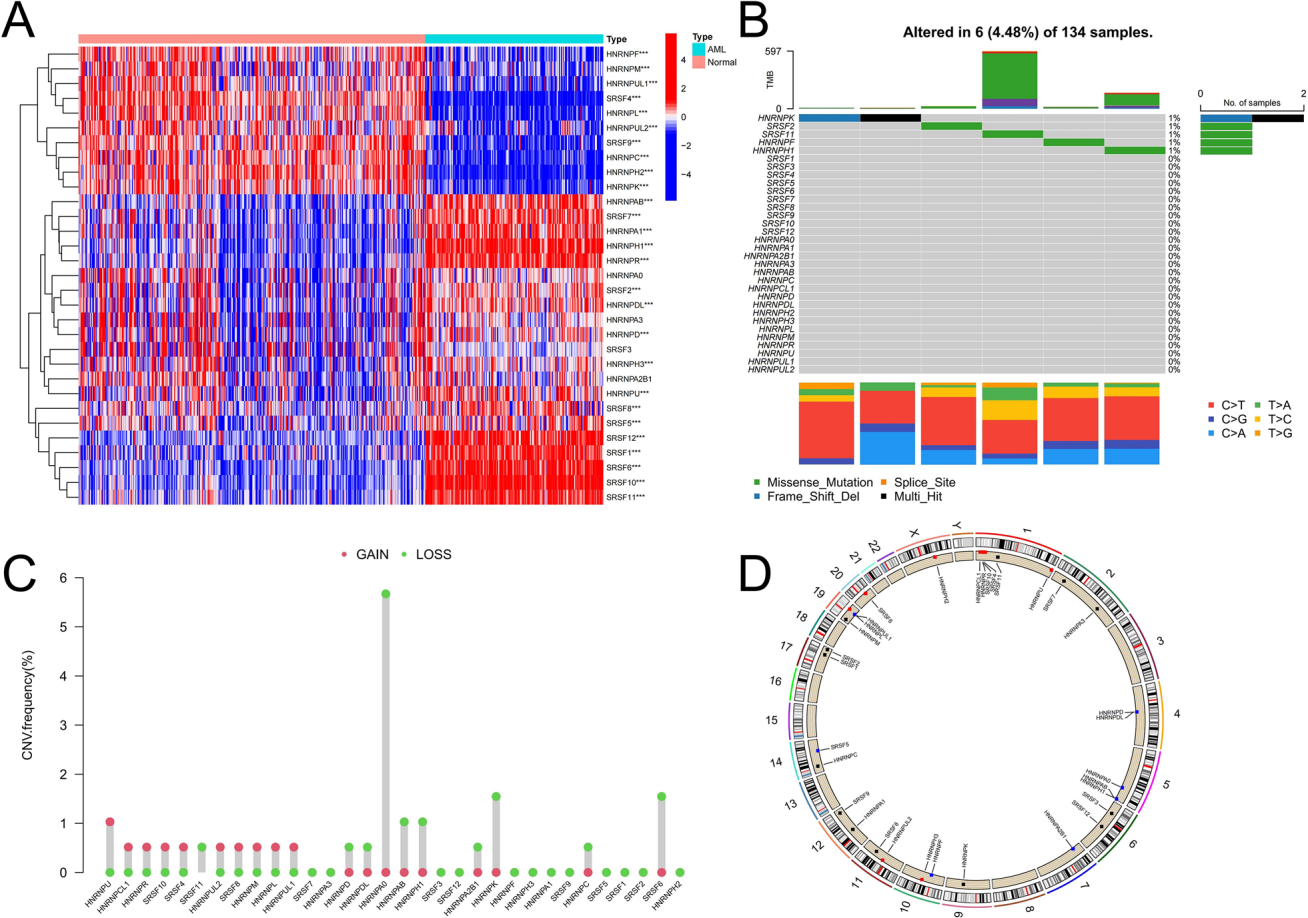
Genetic characteristics of SFs in hnRNP and SR families in AML samples. **A** The heatmap depicts the difference in SFs expression between AML samples and normal samples. **B** Somatic mutations in SFs in 134 TCGA-LAML patient samples; Each column in the waterfall plot represents the mutation type for each patient, the tumor mutation burden (TMB) for each patient is shown in the top half, the mutation frequency and mutation type ratio of SFs are shown on the right, and the proportion of different base transitions is shown below. **C** Copy number variation frequency of SFs. **D** The position of SFs on 23 chromosomes (*P < 0.05; **P < 0.01; ***P < 0.001)

We first analyzed the expression characteristics of two splicing family regulators in AML samples. The expression of 31 out of the 32 SFs was detected in both AML and normal samples. Compared with normal samples, the expression of 10 SFs (HNRNPH2, SRSF4, HNRNPL, HNRNPC, SRSF9, HNRNPK, HNRNPUL1, HNRNPF, HNRNPUL2, HNRNPM) was significantly down-regulated in AML samples. The expression of 17 SFs (HNRNPD, HNRNPH3, SRSF8, HNRNPU, SRSF5, SRSF2, HNRNPDL, HNRNPAB, SRSF7, HNRNPA1, SRSF1, HNRNPR, SRSF6, SRSF11, HNRNPH1, SRSF10, SRSF12) was up-regulated in AML samples. The expression of SRSF3, HNRNPA2B1, HNRNPA3, and HNRNPA0 did not demonstrate significant differences (Fig. 1A). We further analyzed their somatic mutation characteristics, and the results showed that the mutation rate of SFs was low in AML samples. Only 6 out of 134 mutations remained changed, and two of the HNRNPK mutations were frameshift DEL and multihit mutations, respectively. Missense mutations were observed in SRSF2, SRSF11, HNRNPF, and HNRNPH1 in four different samples, and the base changes in these mutations primarily involved conversion from C to T (Fig. 1B). We further conducted copy number variation analysis of SFs to explore the relationship between copy number changes and mRNA expression levels. We observed that the frequency of the increase in the copy number of HNRNPU, HNRNPR, SRSF10, and SRSF8 was upregulated, which may be related to the up-regulation of corresponding mRNA levels. The copy number deletion of HNRNPK and HNRNPC may be one of the reasons for their down-regulation in AML samples (Fig. 1C). Figure 1D shows the positions of the 32 SFs in the chromosome. These results suggest that SFs of hnRNP family and SR family exhibit heterogeneous genetic and expression landscapes in AML samples and may be involved in the onset and progression of AML.

### Regulatory patterns of splicing mediated by 32 SFs

The abnormal changes in the genetic and expression of SFs may be constituted as the signatures of the malignant development of AML. In order to better identify the relationship between splicing factors and the biological process of AML, we aimed to elucidate the relationship between SFs and AML based on the overall expression pattern. The correlation in expression and prognostic characteristics of SFs were analyzed. We found that almost all SFs of the SR family were prognostic risk factors, and hazard ratio (HR) > 1 and a high expression of these genes predicted poor prognosis of patients. However, the 20 SFs of hnRNP family comprised 12 risk factors and 8 protective factors (HR < 1) (Additional file 1: Fig. S1A). Survival analysis showed that the expression of 13 SFs was significantly correlated with prognosis (P < 0.05), and patients with high expression of SRSF12 and HNRNPA1 had a better prognosis. Patients with high expression of SRSF4, HNRNPAB, HNRNPH2, HNRNPUL1, HNRNPF, HNRNPC, HNRNPR, SRSF11, HNRNPL, SRSF6, and SRSF1 showed a significantly worse prognosis (Additional file 1: Fig. S1B). Correlation analysis of the co-expression relationships of these genes showed that they were positively correlated (P < 0.001) (Additional file 1: Fig. S1A).

**Fig. 2:**
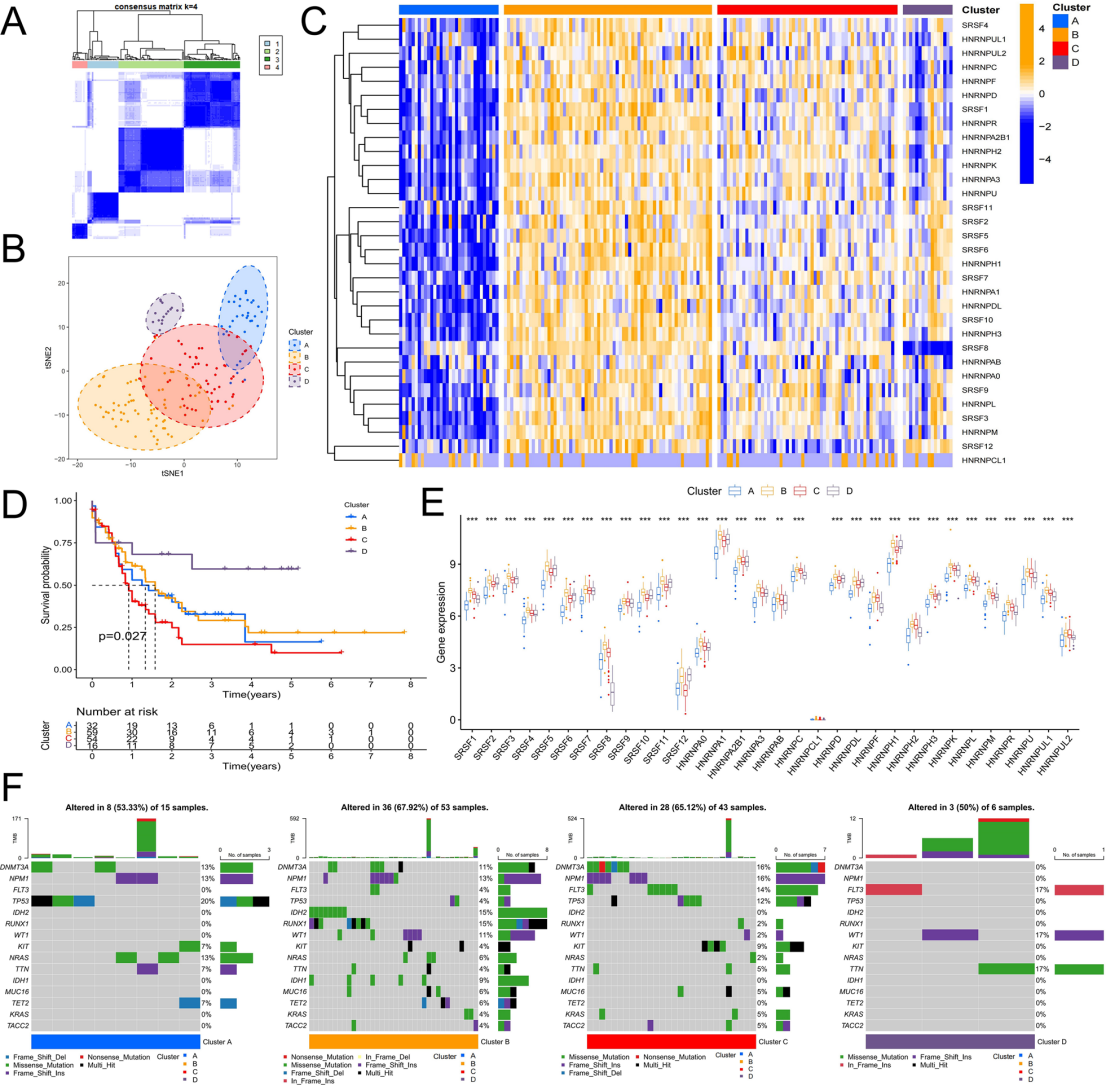
Identification of SFs related regulatory patterns. **A** AML patients were divided into four clusters by consistent clustering algorithm. **B** t-SNE algorithm verifies the clustering ability based on SFs expression. **C** The heatmap shows the expression of SFs in the four clusters. **D** Survival analysis of patients with different clusters. **E** Expression differences of SFs in the four clusters. **F** Differences in somatic mutations among the four clusters (*P < 0.05; **P < 0.01; ***P < 0.001)

We further performed unsupervised clustering based on the expression of SFs. The results showed that patients with AML could be stably divided into four groups, which were termed clusters A–D (Fig. 2A, B). The expression of SFs was generally low in cluster A and high in cluster B, while the overall expression level in cluster C was between that of cluster A and cluster B. The expression of Cluster D was not as uniform as that of the first three clusters (Fig. 2C). Further survival analysis showed that patients in cluster D had the best prognosis, those in cluster C had the worst prognosis, and those in clusters A and B were in the middle (Fig. 2D). Figure 2E further confirms the differential expressed characteristics of SFs in the four clusters. Somatic mutation analysis showed that the TP53 mutation frequency was the highest in cluster A. The percentage of patients with somatic mutations in cluster B was higher, and many patients have multiple mutated genes that may be associated with abnormally activated expression of SFs in this group. The mutant genes in cluster c were concentrated. These genes mainly included *DNMT3A*, *NPM1*, *FLT3*, and *TP53* (Fig. 2F).

### Differences in biological characteristics with respect to different splicing regulation patterns

**Fig. 3:**
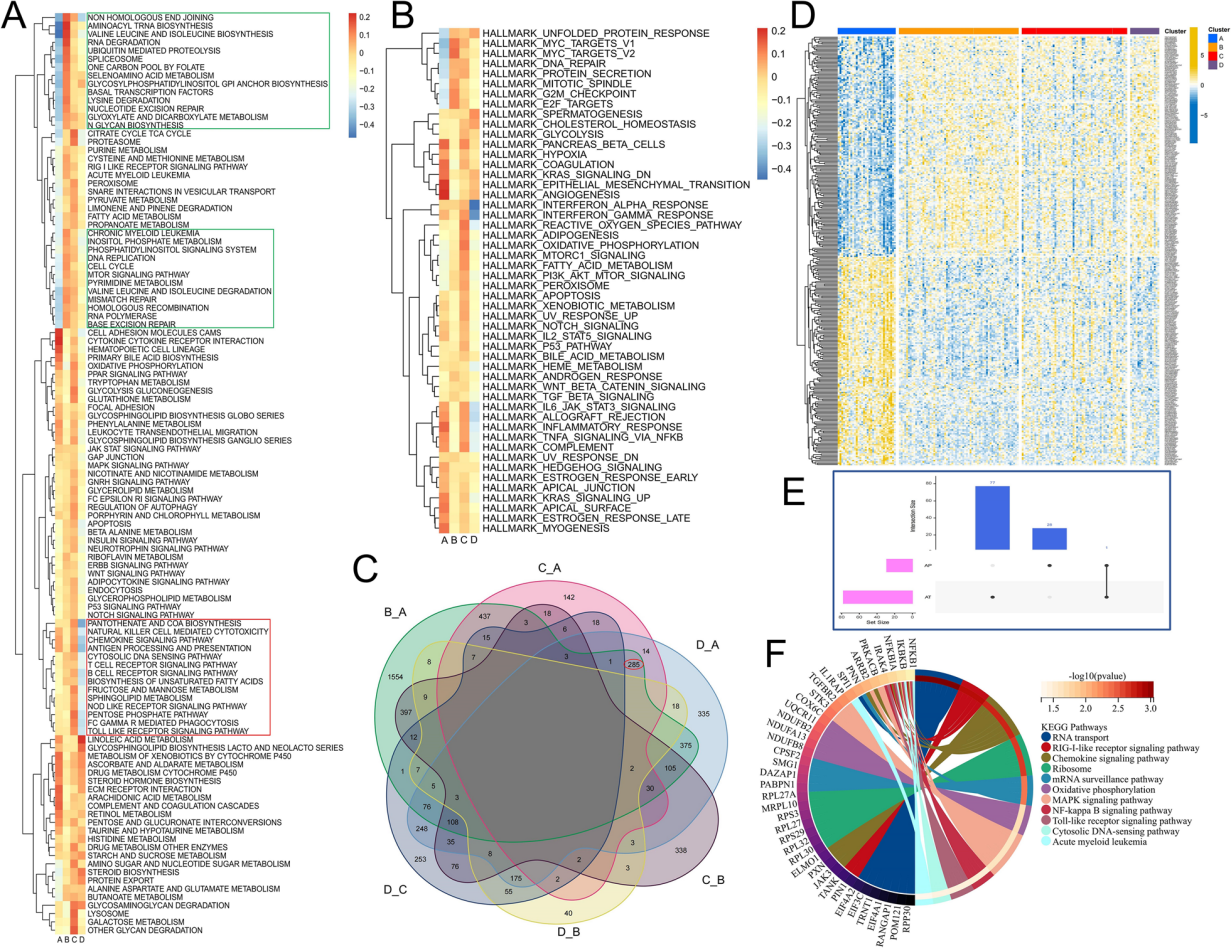
Differences in signaling pathways between different splicing regulation patterns. **A** Difference in enrichment scores of KEGG gene sets related to cancer development in Cluster A and Cluster B, Cluster C and Cluster D. **B** Difference in enrichment scores of cancer-related hallmark gene sets related to cancer development in Cluster A and Cluster B, Cluster C and Cluster D. **C** The Veen diagram shows the intersection of the difference AS events between pairs of comparison in the four clusters. **D** Overlapping AS events after differential expression between cluster A and other clusters. **E** The Upset plot displays the type and number of AS events. **F** Functional analysis of AS event genes

To better analyze the biological differences with respect to splicing regulation patterns, we used the GSVA algorithm to analyze the enrichment differences in KEGG signaling pathways and cancer-related hallmark gene sets associated with different splicing regulation patterns (Fig. 3A, B). In cluster A, the activities of the KRAS signaling pathway mediated up-regulated/down-regulated gene set, IL2/STAT5 signaling pathway, TNF-α signaling pathway via NF-κB, and immune-related pathways, such as coagulation and complement cascade, and the inflammatory response were highly enriched (Fig. 3B), which mainly activates more cell signaling, leading to the continuous release of signals in the pro-cancer pathway. This may be related to a higher proportion of *NRAS* gene mutations in cluster A. Moreover, cluster B showed increased activity of proliferation-related pathways, such as E2F target, cell cycle G2/M checkpoint, MYC-targeted variant 1/2, mitotic spindle, protein regulatory signaling pathways, such as protein secretion and unfolded protein response, and multiple DNA damage repair pathways (Fig. 3A, B). These findings indicate that cluster B showed a significant promotion in cell proliferation, protein expression, and genetic regulation of the genome. Increased activity of DNA damage repair pathways also favors cell survival. Cluster C showed high activity of a large number of immune- and inflammation-related signaling pathways, such as B/T cell receptor signaling pathway, chemokine signaling pathway, NOD-like receptor cell pathway, Toll-like receptor signaling pathway (Fig. 3A), complement cascade, interferon α/γ signaling pathway, IL6-JAK-STAT3 signaling pathway, and inflammatory response (Fig. 3B). Interestingly, the activity of these pathways was lowest in Cluster D. A high activity of these pathways in tumor cells can promote the development of chronic inflammation and immune escape in the tumor microenvironment and may lead to deterioration, which may be an important reason for the poor prognosis of cluster C patients. Cluster C also showed abnormal metabolic reprogramming, with elevated activities of fatty acid metabolism, oxidative phosphorylation, adipogenesis, reactive oxygen species pathway, and heterologous metabolism pathway (Fig. 3B). Abnormal immune and metabolic changes were the main biological characteristics of cluster C.

Apparent differences were observed in biological processes, immune characteristics, and clinicopathological factors among different splicing regulation patterns. We further examined whether different regulation patterns are associated with AS events. We compared four groups pairwise and via veen diagram analysis (Fig. 3C). We found that Cluster A showed the highest number of overlapping differentially expressed AS events with the other three clusters. The heatmap showed that the expression trend of these differential AS events in cluster A was in contrast to that of the other three clusters, with the differences with cluster B being the most apparent (Fig. 3D). We observed that most of the genes in cluster A have a high and a low expressed AS event, which have opposite expression trends, but their splicing types are the same, only the splice sites are different. Further statistical analysis revealed that the splicing types of these events were concentrated at AP and AT (Fig. 3E). AT and AP are different in the last or first exon of the two transcripts, respectively, indicating that different splicing regulation patterns have different effects on the 5′ or 3′ end of the transcript. These differential AS events were observed corresponding to 209 genes. We performed the KEGG enrichment analysis. Results show that these genes were mainly associated with RNA transport, RIG-I-like receptor signaling pathway, Chemokine signaling pathway, ribosome, mRNA surveillance pathway, oxidative phosphorylation, MAPK signaling pathway, NF-kappa B signaling pathway, Toll-like receptor signaling pathway, cytosolic DNA-sensing pathway, and acute myeloid leukemia. Most of these pathways are the same as the significant enrichment pathways of each cluster. SFs are largely involved in the regulation of these AS events. Therefore, the abnormal expression of SFs may be one of the reasons why different clusters have significant differences in biological characteristics.

### Differences in immune-related features among different splicing regulation patterns

**Fig. 4:**
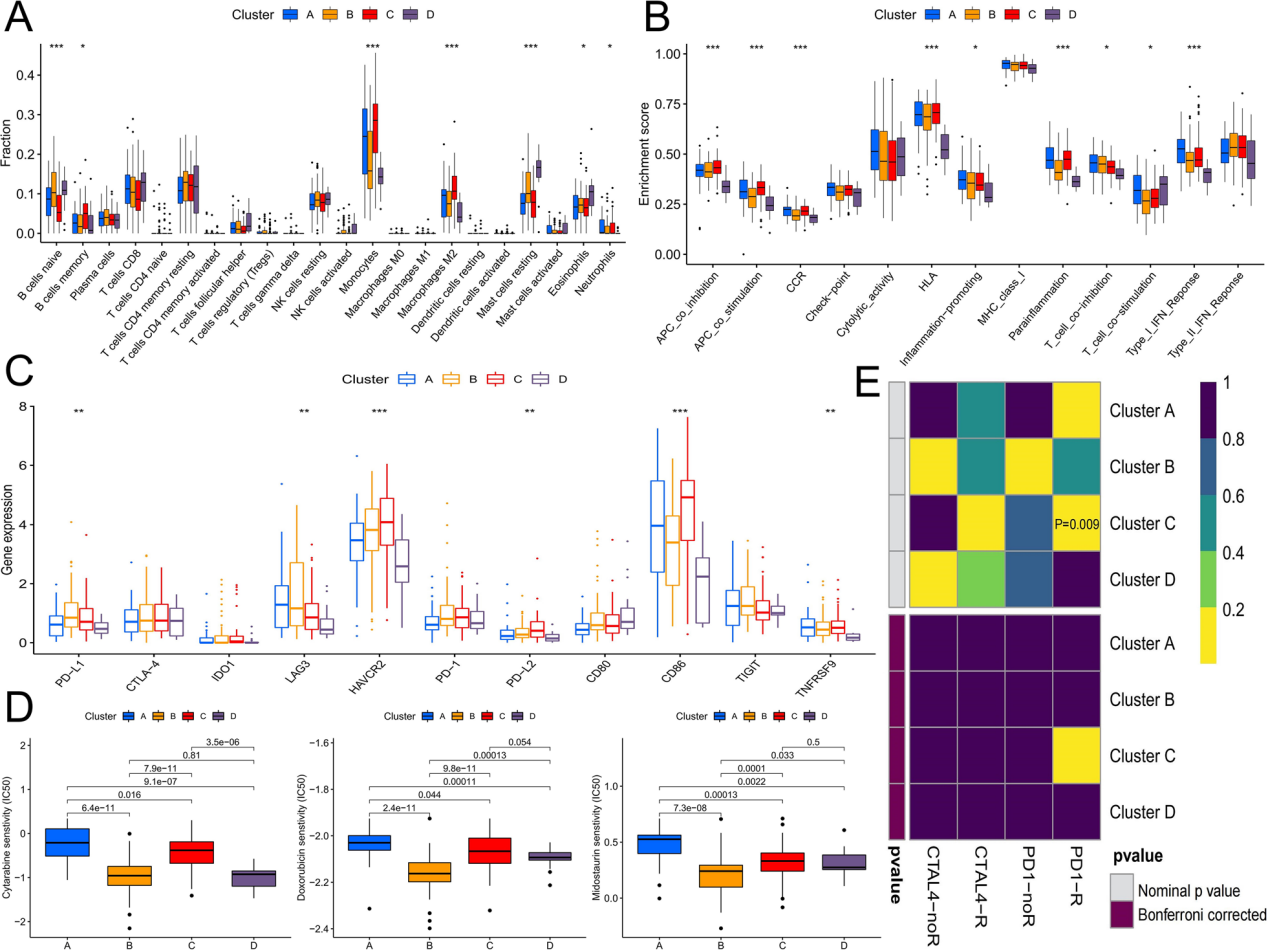
Differences in immune-related features and prediction of treatment sensitivity among different splicing regulation patterns. **A** Differences in infiltration of 22 immune cells among the four clusters. **B** Differences in immune function activity scores among the four clusters. **C** Differences in immune checkpoint expression among the four clusters. **G**, **H** IC50 prediction of the four clusters for cytarabine, doxorubicin, and midostaurin treatment. **I** Prediction of response to anti-PD-1 and anti-CTAL4 immunotherapy by different splicing regulation patterns (*P < 0.05; **P < 0.01; ***P < 0.001)

Pathway enrichment analysis showed significant differences in immune-related pathways among different splicing regulation patterns. We further analyzed the proportion of immune cell infiltration, immune functional activity, and immune checkpoint expression corresponding to different patterns. We observed that cluster C contained more inflammatory immune cells, including monocytes, M2 macrophages, and neutrophils, and the infiltration ratio of CD8+ T cells was the lowest. The infiltration of naive B cells, CD8+ T cells, follicular helper T cells, resting mast cells, and eosinophils was significantly increased in cluster D (Fig. 4A). Cluster A and cluster B showed no significant immune infiltration characteristics. In terms of the expression activity of immune function (Fig. 4B), cluster A showed high activity of antigen-presenting cell (APC) costimulatory molecules, C-C-motif chemokine receptor (CCR), para-inflammation, T-cell costimulatory molecules, and type I interferon (IFN) response. In cluster C, the expression of APC coinhibitory/costimulatory molecules, CCR, and para-inflammation molecules was high. Compared with other clusters, most immune functions were less potent in cluster D, and only T-cell costimulatory molecules show high activity. Differential expression analysis of immune checkpoints showed that the expression levels of HAVCR2, PD-L2, and CD86 in cluster C were significantly higher than those in other clusters (Fig. 4C). Moreover, the overall expression of immune checkpoints was downregulated in cluster D. The characteristics of high infiltration of inflammatory immune cells, high activity of immune cell function, such as APC inhibition, proinflammatory response, and high expression of immune checkpoints in cluster C reflect the possible presence of chronic inflammatory and highly immunosuppressive microenvironment. Hence, these may be the reasons for the poor prognosis of patients in cluster C. In contrast, T cells may play an immune role in cluster D, which may be the reason why patients in cluster D had a better prognosis.

### Prediction of therapeutic sensitivity with different splicing regulation patterns

We predicted the therapeutic sensitivity of common AML drugs based on global gene expression with different splicing regulation patterns. These drugs included cytarabine, doxorubicin, and midostaurin, the first two of which are chemotherapy drugs, and the last one is a targeting agent of *FLT3* gene mutation. The results showed that the IC50 of the three drugs was the highest in cluster A and the lowest in cluster B, and cluster D exhibited higher sensitivity to cytarabine than the other three clusters (Fig. 4D). We further compared the four splicing regulation patterns with the expression dataset of patients with melanoma who responded to immunotherapy, notably the potential therapeutic value of anti-PD-1 treatment for cluster C patients (Fig. 4E).

### Construction and validation of prognostic risk score model

**Fig. 5:**
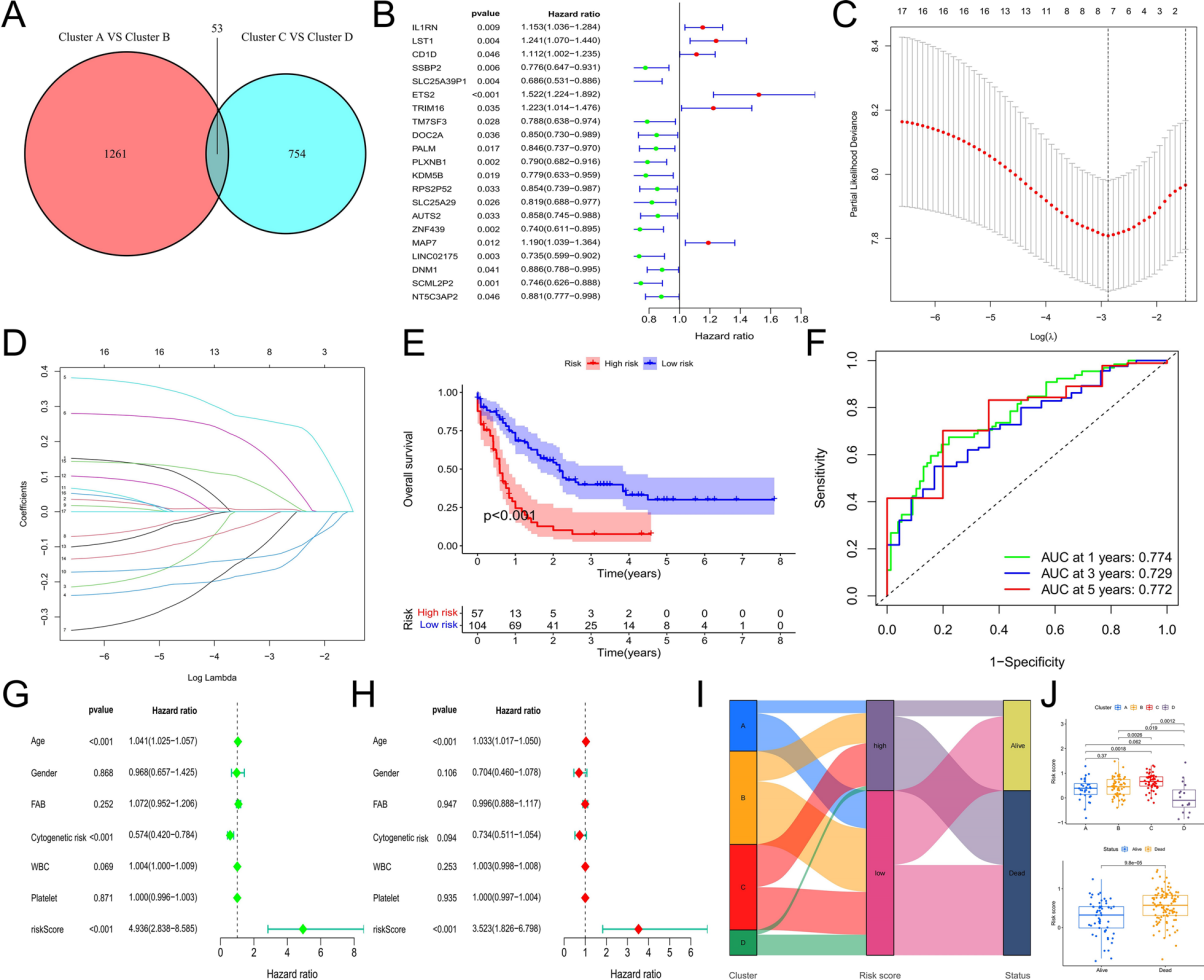
Construction of risk scoring model. **A** Identification of DEGs with different splicing regulation patterns. **B** Identification of DEGs significantly associated with prognosis by Cox regression analysis. **C** Calculate log(λ) of the minimum tenfold cross-validation error point and determine the corresponding model gene. **D** Determine the coefficients of model genes. **E** Survival analysis between high-risk score and low-risk score subgroups. **F** Time-dependent ROC curve analysis of risk score. **G** Univariate Cox regression analysis of clinicopathological factors and risk score. **H** Multivariate Cox regression analysis of clinicopathological factors and risk score. **I** Alluvial diagram showing the changes of splicing regulation patterns, risk score groups, survival status groups. **J** Differences in risk scores among different splicing regulation patterns and survival status groups

Splicing regulation patterns reveal the pathological characteristics and potential treatment modalities of patients with AML, and we further explored their prognostic value. The expressed difference of SFs between cluster A and cluster B was the largest, and the prognosis of patients in custer C and cluster D was significantly different. We performed differential expression analysis for cluster A and cluster B, cluster C and cluster D, and identified 1261 and 754 DEGs (|logFC| > 1, adjusted P value < 0.05), respectively, of which 53 contains both in two groups of DEGs (Fig. 5A). We believe that these genes are closely related to SFs and the prognosis of AML. Cox regression analysis showed that 21 genes were significantly associated with the prognosis of patients with AML (P < 0.05) (Fig. 5B). These prognostic genes were used to construct the prognostic risk model. To prevent overfitting of the model, LASSO regression analysis was used to reduce their dimensionality and eliminate redundant prognostic genes. After tenfold cross-validation, we determined the penalty parameter (λ) of the model and the corresponding eight genes involved in model construction (Fig. 5C), *LST1*, *SSBP2*, *ETS2*, *TRIM16*, *TM7SF3*, *PLXNB1*, *AUTS2*, and *MAP7*. We identified the corresponding correlation coefficients of model genes according to the λ value (Fig. 5D) (Additional file 2: Table S1). Finally, we calculated the risk score for each sample using the model formula. Based on the cut-off value, patients with AML were divided into high-risk and low-risk groups. Log-rank test results showed that the prognosis of patients in the high-risk group was significantly worse than that in the low-risk group (P < 0.001) (Fig. 5E). The time-dependent ROC curve analysis showed that the AUC values of the risk scores in predicting the 1-year, 3-year and 5-year overall survival (OS) of patients with AML were 0.774, 0.729, and 0.772, respectively, indicating that the prognostic risk score model had high predictive accuracy (Fig. 5F). Univariate and multivariate Cox analysis showed that the risk score could be used as an independent prognostic factor (P < 0.001) (Fig. 5G, H). Attributable changes in patients with AML were visualized using the alluvial diagram (Fig. 5I). The risk score further quantified the prognostic characteristics of different splicing regulation patterns. For example, cluster C had the worst prognosis and the highest risk score, while cluster D showed contrasting results (Fig. 5J). Meanwhile, significant differences in risk scores were observed among patients with different survival status (Fig. 5J).

**Fig. 6:**
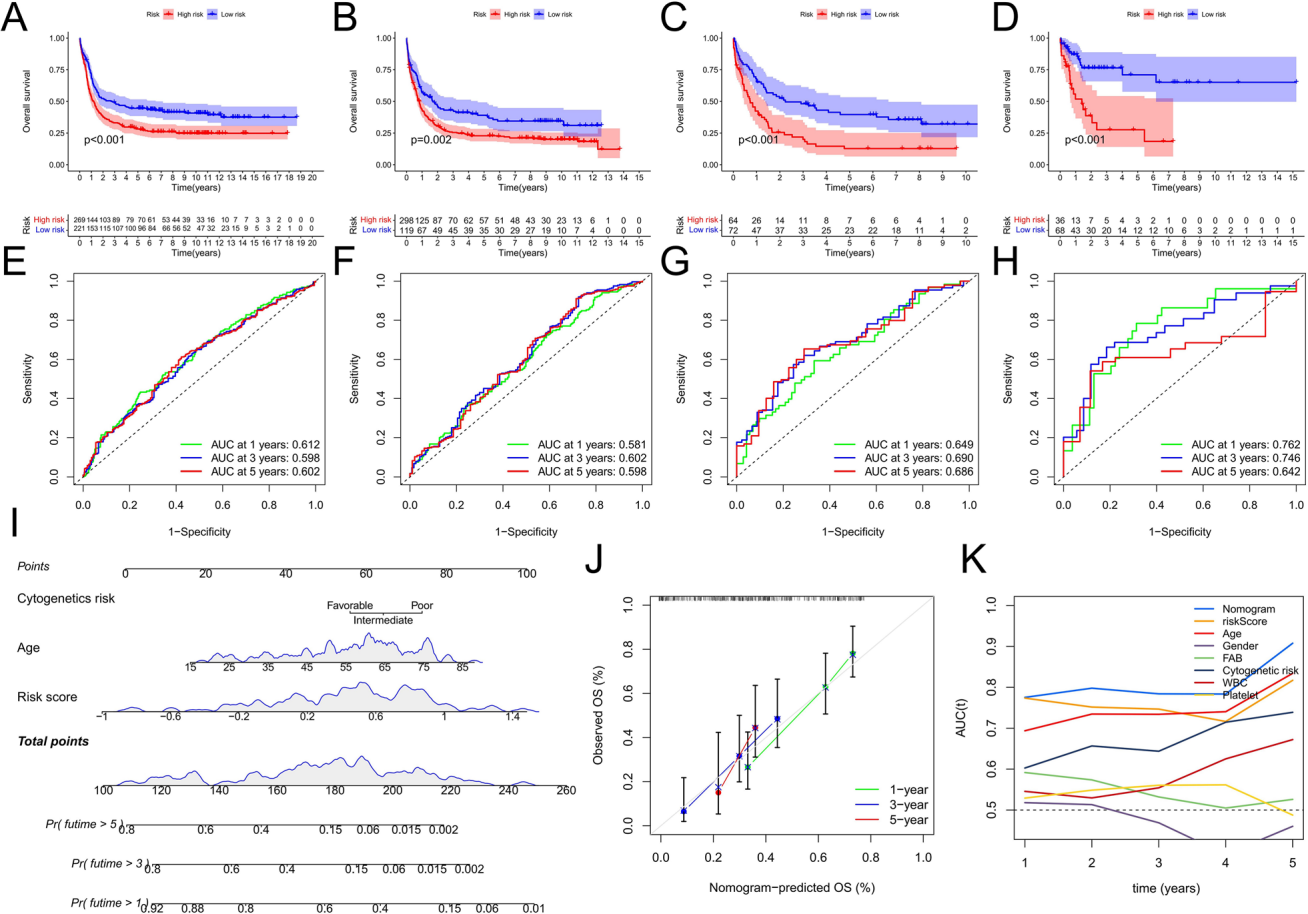
Validation of risk score model and construction of nomogram. **A**–**D** Survival analysis between the high- and low-risk score groups in the Validation cohorts. **A** GSE14468; **B** GSE37642-GPL96; **C** GSE37642-GPL570; **D** GSE71014. Log-rank test. **E**–**H** Time-dependent ROC curve analysis of the risk score in the Validation cohorts. **E** GSE14468; **F** GSE37642-GPL96; **G** GSE37642-GPL570; **H **GSE71014. **I** Nomogram to predict OS in AML patients. **J** Time-dependent calibration curve to validate the predictive power of the nomogram. **K** ROC curve analysis of nomogram and other prognostic factors

Next, four validation cohorts confirmed the prognostic value of the risk score. The OS of patients with high-risk scores was significantly shortened (Fig. 6A–D), and the ROC curve also indicated the predictive robustness of the risk score model (Fig. 6E–H).

### Construction of a nomogram to predict OS

In order to predict the OS of patients more accurately, we combined the clinicopathological factors (age and cytogenetic risk) significantly related to the prognosis of patients with AML with the risk score model to construct a nomogram (Fig. 6I). By calculating the total score of each patient in the nomogram, the corresponding 1-, 3-, and 5-year survival rates were observed. The 1-, 3-, and 5-year calibration curves also proved that nomograms could accurately predict OS (Fig. 6J). The time-dependent ROC curve showed that the nomogram had the highest AUC value (Fig. 6K). Taken together, these results indicate that the nomogram we constructed can further improve the accuracy of OS prediction in patients with AML. Furthermore, it also provides a new method for clinical prognosis evaluation.

### Expression of the splicing factor SRSF10 was up-regulated in AML

**Fig. 7:**
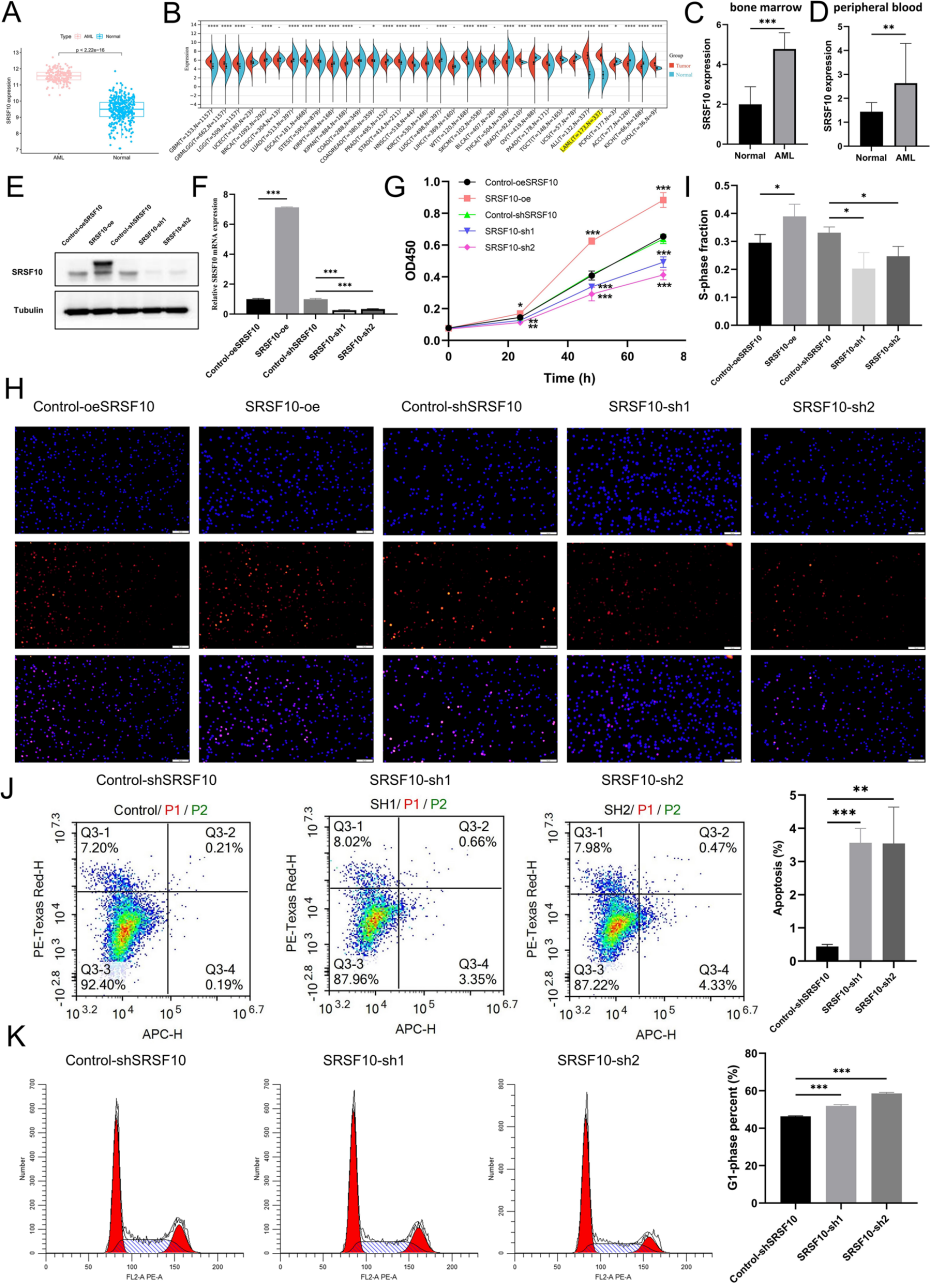
Expression characteristics of SRSF10 in AML and its relationship with malignant phenotypes of AML cells. **A** Differences in mRNA expression of SRSF10 between TCGA-LAML samples and GTEx normal blood samples. **B** Differences in mRNA expression of SRSF10 between 34 cancer samples and normal control samples in GDC database and GTEx database, with AML in yellow. **C** Differences in mRNA expression of SRSF10 between bone marrow (BM) samples from 4 AML patients and peripheral blood (PB) samples from 8 healthy controls. **D** Differences in mRNA expression of SRSF10 in peripheral blood samples from 22 AML patients and 23 healthy controls. **E**, **F** mRNA and protein expression levels of SRSFF10 in THP-1 cells in SRSF10 overexpression group (SRSF10-oe) and Control group (Control-oeSRSF10), and two knockdown groups (SRSF10-SH1, SRSF10-SH2) and Control group (Control-shSRSF10). **G** Absorbance at 450 nm wavelength after CCK8 treatment in different SRSF10 treatment groups at different time nodes. The more absorbance increased, the more cell proliferation. **H** EdU staining was performed on different SRSF10 treatment groups. Top to bottom were all cells in the field of view, S-phase proliferating cells, and the composite of the above two images. The more pink cells, the more proliferating cells. **I** The ratio of cells in proliferative phase to all cells in a single field observed by fluorescence microscopy after EdU staining in different SRSF10 treatment groups. The larger the ratio, the more cells in proliferative phase. **J** Apoptosis levels in different SRSF10 treatment groups. **K** Cell cycle changes in different SRSF10 treatment groups (*P < 0.05; **P < 0.01; ***P < 0.001)

Among the 32 SFs, we observed that the expression of SRSF12 and SRSF10 was most significantly upregulated in AML. Based on the low expression of SRSF12 and its prognostic protection factor, we only conducted an in-depth study on the risk factor SRSF10 to explore its relationship with the onset and development of AML. The up-regulated expression of SRSF10 in AML samples of the TCGA database and normal blood samples of the GTEx database is shown in Fig. 7A. Meanwhile, we analyzed the expression level of SRSF10 in the pan-cancer atlas of the TCGA database and found that the expression of SRSF10 was most up-regulated in AML and acute lymphoblastic leukemia (ALL), indicating that it may be involved in hematological tumorigenicity and development (Fig. 7B). We further verified the expression of SRSF10 in AML clinical samples. PCR results showed that the expression of SRSF10 in AML bone marrow samples and peripheral blood samples was significantly higher than that in normal control samples (Fig. 7C, D).

### Splicing factor SRSF10 plays a cancer-promoting role in AML cells

To further clarify the biological role of SRSF10 in the development of AML, we obtained cDNA containing SRSF10 sequence and a plasmid targeting shRNA, which were packaged with lentivirus, followed by infection of the AML cell line THP-1. Stably transfected cell lines with SRSF10 overexpression (oeSRSF10) and knockdown (shSRSF10) were screened. As shown in Fig. 7E, F, the transfection efficiency was more than 80% and was verified by PCR and WB experiments.

The proliferation of THP-1 cells was detected by CCK8 assay and EdU assay after SRSF10 overexpression and knockdown. CCK8 assay showed that compared with the control-oeSRSF10 group, the proliferation ability of THP-1 cells in the SRSF10-oe group was significantly enhanced. Compared with the control-shSRSF10 group, the proliferation of THP-1 cells in the SRSF10-SH1 and SRSF10-SH2 groups was significantly reduced (Fig. 7G). EdU staining also showed that more cells underwent DNA replication in the SRSF10-oe group than in the control-oeSRSF10 group. However, fewer cells underwent DNA replication in the SRSF10-SH1 and SRSF10-SH2 groups than in the control-ShSRSF10 group (Fig. 7H, I). All these results demonstrated that SRSF10 overexpression promoted THP-1 cell proliferation, while SRSF10 knockdown inhibited THP-1 cell proliferation.

We used Annexin V-FITC/PI staining to label apoptotic cells. Apoptosis in control-shSRSF10, SRSF10-SH1, and SRSF10-SH2 groups were detected by flow cytometry. The apoptosis rate of the SRSF10-SH1 and SRSF10-SH2 groups was higher than that of the control group (Fig. 7J). The apoptosis trend was consistent with the results of CCK8 method and EdU staining, which were performed to detect cell proliferation ability in different transfection groups. A stronger proliferation of cells corresponded to weaker apoptosis. We further detected the changes in the cell cycle of THP-1 after SRSF10 knockdown by flow cytometry. The results showed that compared with control-ShSRSF10, the arrest of THP-1 cells in the G1 phase was more apparent in the SRSF10-SH1 and SRSF10-SH2 groups (Fig. 7K).

## Discussion

Examination of the mechanism and treatment of AML remains a challenging issue [20]. Although many studies have revealed the abnormal characteristics and carcinogenic effects of AML with respect to multiple aspects, such as genomic mutation, immunosuppression, and metabolic reprogramming field [21–23], a systematic understanding of the pathogenesis is lacking, which is the goal of all tumor studies. Meanwhile, the drug resistance of treatment is also accompanied by the complex pathological changes in the disease, corresponding to a dilemma of “while the priest climbs a post, the devil climbs ten” [7]. Therefore, it is of great significance to explore new pathogenic mechanisms and therapeutic targets.

The role of abnormal splicing of genes in AML progression and drug resistance has garnered interest, and studies have shown that recurrent SF mutations are important drivers of hematological malignancies [24]. AS is an important component of normal hematopoiesis and is necessary for cell differentiation and rapid response to external stimuli [14]. However, the imbalance in splicing mechanisms can lead to abnormal pathologies related to malignancies such as MDS and AML [25], for instance, affecting the apoptotic sensitivity of AML cells and induction of drug resistance [26]. Patients with AML with the highest SF mutation rate have common ontogenetic characteristics of the disease, one of which is the high incidence of treatment failure [21]. Although splicing classification is not a widely used method of classification or risk stratification in AML, the potential for the analysis of these characteristics required further evaluation. With a growing understanding of AML splicing dysregulation, new therapeutic targets may gradually emerge, and therapeutic strategies that target key spliceosome elements (such as SF3B1) or specific oncogenic splice variants in a manner that circumvents resistance mechanisms may prove invaluable in eradicating these types of AML clones [27].

This study explored SFs with the most extensive and prominent functions in the hnRNP and SR families. Mutations and abnormal expression of SFs can influence the expression changes of different subtypes of many genes, which may have completely different functions. For example, the Bcl-2 family generates pro-apoptotic and anti-apoptotic components through AS [28]. We explored the genomic and transcriptomic changes in 32 SFs from hnRNP and SR families in AML samples from the TCGA database by bioinformatics analysis. Consistent with previous studies, the mutation rate of SFs in patients with AML was not high [17]. However, compared with normal samples, the transcriptome of SFs in AML samples changed significantly, with most SFs showing up-regulated expression. This suggests that the overexpression of SFs may contribute to the development of AML by significantly increasing the splicing during AS events. In subsequent analysis, we also observed that most of the up-regulated SFs, such as HNRNPAB, HNRNPR, SRSF1, SRSF6 and SRSF11, were significantly associated with poor prognosis in patients with AML. As an oncogenic SF, SRSF1 can regulate the splicing of several proteins in the apoptotic pathway, including Bcl-2 like protein 11 (BIM)-promoting AS, producing subtypes lacking pro-apoptotic function [29]. Overexpression of SRSF1 can induce tumor formation in epithelial cells and inhibit apoptosis of breast cancer cells [29]. In addition, SRSF1 expression is associated with mTORC1 activation [30], a signaling pathway associated with AML progression and clonal selection during minimal residual disease repropagation [31].

Tumor classification is a method used to distinguish the degree of malignancy and heterogeneity of tumors. Classification according to the individual characteristics of different patients is helpful for clinical diagnosis, treatment, and prognosis evaluation. We performed a clustering analysis based on the expression of SFs and identified four splicing regulation patterns in different patients with AML. Moreover, the biological processes of these four splicing regulation patterns are significantly different. In the pattern with generally low expression of SFs, a variety of oncogenic signal transduction pathways are significantly activated, such as KRAS, IL2/STAT5, NF-κB/TNF-α, and WNT-β/catenin pathways. Therefore, the typically low expression of SFs can be used as a signature to indicate abnormal changes in the oncogenic pathway. However, in the pattern with high expression of SFs, we observed a significant enhancement of cell proliferation signals, both the cell cycle-related pathways and the expression of genetic information, showing a highly active state, which can also be used as a signature of disease development. It is well understood that SFs are themselves involved in the regulation of transcriptome, and their high expression and proliferation-induction activity are mutually promoting. In addition, with potent cell life activities, this splicing regulation pattern also fully activates DNA damage repair-related signal pathways to ensure the normal expression of genetic information. The expression of SFs in the other two splicing regulation patterns was not as consistent as the first two patterns, but the difference in prognosis between the two groups was the most apparent. When the expression level of SFs is between the first two patterns, the prognosis of patients was the worst. In this splicing regulation pattern, we observed a significant increase in the infiltration rate of inflammatory immune cells such as monocytes, M2 macrophages, and neutrophils, and a high expression level of immune checkpoints, suggesting a tumor microenvironment with chronic inflammatory development and immunosuppression, corresponding to various pro-inflammatory and immune-related signaling pathways, such as B-cell receptors, chemokines, NOD-like receptors, and Toll-like receptors signaling pathways. In the splicing regulation pattern with the best prognosis, most of the prognostic risk-associated SFs were underexpressed, while SFs with protective effects and association with a positive prognosis, such as SRSF12 and HNRNPH1, were significantly overexpressed. These expressive features indicate the benefits of survival. In this pattern, the anti-tumor immunity mediated by T cells may play an important role, which is more conducive to the survival of patients.

Splicing regulation patterns can be used for tumor classification to better elucidate the pathological status and tumor microenvironment characteristics of patients with AML. To better guide the development of personalized treatment plans and prognosis evaluations of patients with AML, we also predicted the treatment sensitivity of patients with different splicing regulation patterns. Patients with splicing regulation patterns with generally high expression of SFs were more sensitive to cytarabine, doxorubicin, and midostaurin. Patients with the best survival splicing regulation pattern also benefited from cytarabine. In the prediction of immunotherapy response, patients with splicing regulation pattern with moderate expression level of SFs and showing the worst prognosis had a higher response to anti-PD-1 treatment. In addition, the prognostic risk score model can accurately predict the prognosis of patients with AML. The nomogram constructed by combining age and cytogenetic risk can better predict the OS of patients, which can be used as a prognostic prediction tool to help clinicians better evaluate the prognosis of patients.

Finally, we investigated the cancer-promoting mechanism of SRSF10 in AML. SRSF10 is highly expressed in many cancers and plays a cancer-promoting role. For instance, it promotes the production of the cancer-promoting splice variant BCLAF1-L [32]. It regulates the alternative splicing of cancer-related transcripts MDM4 and SLK in HCT116 cells [33]. SRSF10 upregulates the production of a circular RNA (CIRC-ATXN1) that plays a role in glioma angiogenesis by sequestrating mir-526b-3p, which normally inhibits the expression of pro-angiogenic MMP2 and VEGFA [34]. Our study showed that the knockdown of SFSF10 could inhibit the proliferation of AML cells and increase the apoptosis, and more cells were arrested in G1 phase compared with the control group. Overexpression of SRSF10 significantly promoted the malignant phenotype of AML cells, indicating that SRSF10 promoted the development of AML. A previous study identified SRSF10 as an important RNA binding protein for AML cell survival through CRISPR/Cas9 technology. Our experiment also confirmed that SRSF10 can promote the malignant phenotype of AML cells [35]. Therefore, SRSF10 can be used as a potential biomarker for AML. It also provides a new direction to elucidate the mechanism of AML from the perspective of AS.

In summary, this study analyzed the expression landscape of SF families represented by hnRNP and SR through multi omics data. The splicing regulation patterns identified based on SF expression are significantly different in many biological processes and immune characteristics. The recognition of these patterns has potential reference value for the evaluation of tumor microenvironment and clinical results of patients with AML. These findings provide a new perspective for the establishment of personalized therapy. However, this study also has some limitations. First, there is a lack of larger sample data and clinical reality cohort to verify the characteristics of splicing regulation patterns. Second, we only explored the relationship between SRSF10 and the malignant phenotype of AML cells. More efforts are still needed to explore broader SFs and more detailed splicing regulation mechanisms.

## Conclusion

This study revealed the expression characteristics of hnRNP and SR families SFs in AML, as well as the differences in biological processes and clinicopathological factors of different splicing regulation patterns. The analysis based on splicing regulation patterns can promote our understanding of the relationship between tumor microenvironment and AS in AML, which can aid in the development of clinical personalized treatment plans. Meanwhile, the prognostic risk score model can accurately predict the prognosis of patients with AML. In subsequent experimental analysis, we confirmed that SRSF10 promoted the development of malignant phenotype in AML cells, suggesting that SRSF10 could be used as a potential therapeutic target and biomarker for AML.

## Supplementary Information

**Additional file 1: Figure S1.** Correlation analysis of splicing factor expression and prognosis of AML patients (**A**), Kaplan-Meier curve analysis between groups with high and low expression of splicing factor (**B**).

**Additional file 2: Table S1.** Genes and coefficients of risk score model.

## Data Availability

The datasets generated and/or analyzed during the current study available from the corresponding author on reasonable request.

## Abbreviations

AS: Alternative splicing
AML: Acute myeloid leukemia
SF: Splicing factor
hnRNP: Heterogeneous nuclear ribonucleoprotein
SR: Serine/arginine-rich
pre-mRNA: Precursor messenger RNA
TCGA: The Cancer Genome Atlas
GTEx: Genome Tissue Expression
GEO: Gene Expression Omnibus
KEGG: Kyoto Encyclopedia of Genes and Genomes
GO: Gene Ontology
GSEA: Gene set enrichment analysis
DEG: Differentially expressed gene

## References

[1] Short N, Rytting M, Cortes J (2018). Acute myeloid leukaemia. Lancet.

[2] Juliusson G (2009). Age and acute myeloid leukemia: real world data on decision to treat and outcomes from the Swedish acute leukemia registry. Blood.

[3] DiNardo C, Perl A (2019). Advances in patient care through increasingly individualized therapy. Nat Rev Clin Oncol.

[4] DiNardo C (2019). Venetoclax combined with decitabine or azacitidine in treatment-naive, elderly patients with acute myeloid leukemia. Blood.

[5] Wei A (2019). Venetoclax combined with low-dose cytarabine for previously untreated patients with acute myeloid leukemia: results from a phase Ib/II study. J Clin Oncol.

[6] Rivera OD (2021). Alternative splicing redefines landscape of commonly mutated genes in acute myeloid leukemia. Proc Natl Acad Sci USA.

[7] de Necochea-Campion R, Shouse GP, Zhou Q, Mirshahidi S, Chen CS (2016). Aberrant splicing and drug resistance in AML. J Hematol Oncol.

[8] Adamia S (2014). NOTCH2 and FLT3 gene mis-splicings are common events in patients with acute myeloid leukemia (AML): new potential targets in AML. Blood.

[9] Bose P, Grant S (2013). Mcl-1 as a therapeutic target in acute myelogenous leukemia (AML). Leuk Res Rep.

[10] Mohamed AM (2016). Oncogene- and drug resistance-associated alternative exon usage in acute myeloid leukemia (AML). Oncotarget.

[11] Zhang QX (2022). Alternative splicing analysis showed the splicing factor polypyrimidine tract-binding protein 1 as a potential target in acute myeloid leukemia therapy. Neoplasma.

[12] Lee Y, Rio DC (2015). Mechanisms and regulation of alternative pre-mRNA splicing. Annu Rev Biochem.

[13] Ray D (2013). A compendium of RNA-binding motifs for decoding gene regulation. Nature.

[14] Grech G (2014). Expression of different functional isoforms in haematopoiesis. Int J Hematol.

[15] Zhang J, Manley JL (2013). Misregulation of pre-mRNA alternative splicing in cancer. Cancer Discov.

[16] Makishima H (2012). Mutations in the spliceosome machinery, a novel and ubiquitous pathway in leukemogenesis. Blood.

[17] Karimi M (2015). High-throughput mutational screening adds clinically important information in myelodysplastic syndromes and secondary or therapy-related acute myeloid leukemia. Haematologica.

[18] Adamia S (2014). A genome-wide aberrant RNA splicing in patients with acute myeloid leukemia identifies novel potential disease markers and therapeutic targets. Clin Cancer Res.

[19] Charoentong P (2017). Pan-cancer immunogenomic analyses reveal genotype–immunophenotype relationships and predictors of response to checkpoint blockade. Cell Rep.

[20] DiNardo CD, Perl AE (2019). Advances in patient care through increasingly individualized therapy. Nat Rev Clin Oncol.

[21] Lindsley RC (2015). Acute myeloid leukemia ontogeny is defined by distinct somatic mutations. Blood.

[22] Tettamanti S, Pievani A, Biondi A, Dotti G, Serafini M (2021). Catch me if you can: how AML and its niche escape immunotherapy. Leukemia.

[23] Kreitz J (2019). Metabolic plasticity of acute myeloid leukemia. Cells.

[24] Hahn CN, Venugopal P, Scott HS, Hiwase DK (2015). Splice factor mutations and alternative splicing as drivers of hematopoietic malignancy. Immunol Rev.

[25] Larsson CA, Cote G, Quintás-Cardama A (2013). The changing mutational landscape of acute myeloid leukemia and myelodysplastic syndrome. Mol Cancer Res.

[26] Dubrez L, Berthelet J, Glorian V (2013). IAP proteins as targets for drug development in oncology. Onco Targets Ther.

[27] Bonnal S, Vigevani L, Valcárcel J (2012). The spliceosome as a target of novel antitumour drugs. Nat Rev Drug Discov.

[28] Akgul C, Moulding DA, Edwards SW (2004). Alternative splicing of Bcl-2-related genes: functional consequences and potential therapeutic applications. Cell Mol Life Sci.

[29] Anczuków O (2012). The splicing factor SRSF1 regulates apoptosis and proliferation to promote mammary epithelial cell transformation. Nat Struct Mol Biol.

[30] Karni R, Hippo Y, Lowe SW, Krainer AR (2008). The splicing-factor oncoprotein SF2/ASF activates mTORC1. Proc Natl Acad Sci USA.

[31] Hoshii T (2012). mTORC1 is essential for leukemia propagation but not stem cell self-renewal. J Clin Invest.

[32] Zhou X (2014). BCLAF1 and its splicing regulator SRSF10 regulate the tumorigenic potential of colon cancer cells. Nat Commun.

[33] Sohail M (2021). A novel class of inhibitors that target SRSF10 and promote p53-mediated cytotoxicity on human colorectal cancer cells. NAR Cancer.

[34] Arber D (2016). The 2016 revision to the World Health Organization classification of myeloid neoplasms and acute leukemia. Blood.

[35] Wang E (2019). Targeting an RNA-binding protein network in acute myeloid leukemia. Cancer Cell.

