# Supplementary figures and images for: Splicing factor-mediated regulation patterns reveals biological characteristics and aid in predicting prognosis in acute myeloid leukemia
Source: J Transl Med. 2023 Jan 7;21:6. doi: 10.1186/s12967-022-03868-9 (PMC9824960; doi:10.1186/s12967-022-03868-9)

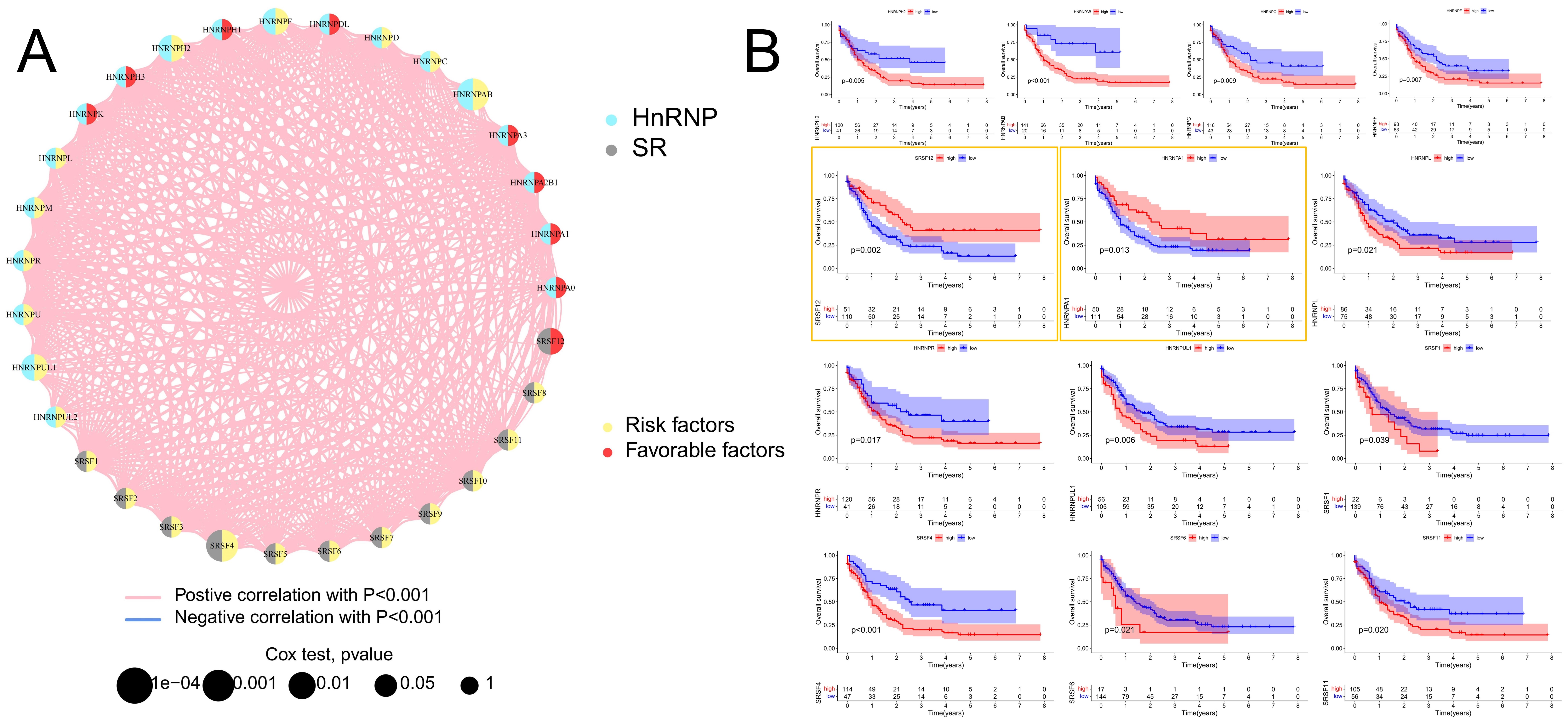

Supplement: Supplementary file 1 — Additional file 1: Figure S1. Correlation analysis of splicing factor expression and prognosis of AML patients (A), Kaplan-Meier curve analysis between groups with high and low expression of splicing factor (B). [file 12967_2022_3868_MOESM1_ESM.jpg]
